# Strategies for Imaging Androgen Receptor Signaling Pathway in Prostate Cancer: Implications for Hormonal Manipulation and Radiation Treatment

**DOI:** 10.1155/2013/460546

**Published:** 2013-10-29

**Authors:** Gravina Giovanni Luca, Claudio Festuccia, Pierluigi Bonfili, Mario Di Staso, Pietro Franzese, Valeria Ruggieri, Vladimir M. Popov, Vincenzo Tombolini, Carlo Masciocchi, Eleonora Carosa, Andrea Lenzi, Emmanuele A. Jannini, Ernesto Di Cesare

**Affiliations:** ^1^Department of Applied, Clinical and Biotechnological Sciences, Laboratory of Radiobiology and Division of Radiotherapy, University of L'Aquila, Via Vetoio, Coppito 2, L'Aquila, Italy; ^2^Department of Experimental Medicine, Section of Medical Pathophysiology, Food Science and Endocrinology, Sapienza University of Rome, Italy; ^3^LIPOGEN LLC, Mount Laurel, NJ, USA; ^4^Division of Radiotherapy, University of Rome, University Hospital “Policlinico Umberto I”, Italy; ^5^Department of Radiological Sciences, Division of Radiotherapy, University of Rome “La Sapienza”, Spencer-Lorillard Foundation, Rome, Italy; ^6^Department of Applied, Clinical and Biotechnological Sciences, Division of Radiology, University of L'Aquila, L'Aquila, Italy; ^7^Course of Endocrinology and Medical Sexology, Department of Applied, Clinical and Biotechnological Sciences, University of L'Aquila, L'Aquila, Italy

## Abstract

Prostate cancer (Pca) is a heterogeneous disease; its etiology appears to be related to genetic and epigenetic factors. Radiotherapy and hormone manipulation are effective treatments, but many tumors will progress despite these treatments. Molecular imaging provides novel opportunities for image-guided optimization and management of these treatment modalities. Here we reviewed the advances in targeted imaging of key biomarkers of androgen receptor signaling pathways. A computerized search was performed to identify all relevant studies in Medline up to 2013. There are well-known limitations and inaccuracies of current imaging approaches for monitoring biological changes governing tumor progression. The close integration of molecular biology and clinical imaging could ease the development of new molecular imaging agents providing novel tools to monitor a number of biological events that, until a few years ago, were studied by conventional molecular assays. Advances in translational research may represent the next step in improving the oncological outcome of men with Pca who remain at high risk for systemic failure. This aim may be obtained by combining the anatomical properties of conventional imaging modalities with biological information to better predict tumor response to conventional treatments.

## 1. Molecular Imaging as Tool for Translating Biological Information into Oncological Practice

Pca is one of the most commonly diagnosed cancers in men. Its etiology appears to be related to lifestyle patterns, genetic, epigenetic factors, and hormones [1, 2]. Surgery and external beam radiation therapy (EBRT) remain to be two of the major milestones for the treatment of localized or locally advanced Pca [3]. Despite their technical improvements, Pca recurrence is not uncommon [3] after these treatments. Historically, hormonal manipulation has been used for the management of advanced and/or recurrent Pca [3] especially in association with radiotherapy [4]. We now have a greater understanding of mechanisms sustaining CRPC upon hormonal manipulation [5]. Biological evidence supports the idea that androgen receptor (AR) drives the transition towards an androgen independent and radioresistant phenotype [6]. However, we are aware that the improvement in oncological outcome of men who remain at high risk for systemic failure may also be achieved by improving the diagnostic performances of conventional imaging modalities by making them more suitable for predicting tumor response to conventional treatments. There are well-known limitations and inaccuracies in current imaging approaches for monitoring biological changes governing tumor progression and radioresistant phenotypes [7]. The growing number of alternative treatments and the need for an early identification of nonresponders have considerably stimulated and renewed the interest to use molecular imaging techniques [7]. The close integration between molecular biology and clinical imaging may ease the development of new molecular imaging agents useful to monitor a number of biological events that, until a few years ago, were studied by conventional molecular assays [8]. With regard to Pca, progress in quantification, characterization, and timing of biological processes could create novel opportunities to more fully characterize many biological events and to monitor the performance of well-established as well as novel treatment modalities [8]. However, there are theoretical and practical challenges in attempting to translate these imaging strategies into clinical practice [9]. Some of these challenges include the need to overcome problems related to the amplification of low level signals of *in vivo* biological events, the development of integrated imaging platforms with sufficiently high spatial and temporal resolution [9], and the need to reach the target *in vivo* to achieve satisfactory specificity [7–9]. The advances in the molecular based approaches in radiology are specifically evident in oncological treatments [10]. One of the most striking examples of foregoing statements is attested by the development of the enormous amount of specific drugs and inhibitors, the ability to genetically modify cellular systems, and the introduction of a multitude of diagnostic tools able to monitor individual molecular and biological processes [8]. These achievements have dramatically augmented our understanding of molecular oncology and this body of knowledge can now be translated into new drugs or agents for molecular imaging by allowing detection of patients with specific molecular profiles and improving patient care [11]. However the question of whether radiology will be able to integrate the molecular imaging into the mainstream molecular research and really translate biological knowledge and discoveries into clinical practice is still open. Historically, although most radiological research has been focused on the attempt to improve technological quality, substantial advances in MR spectroscopy, diffusion weighted imaging, dynamic contrast enhanced methods, and contrast agents as well as radiochemistry advances in tumor targeting agents such as antibodies and PET radiopharmaceuticals have greatly improved the overall diagnostic performance [8–11]. Today's medical imaging technology has improved significantly in terms of resolution and speed reaching a stage where cell trafficking can be efficiently imaged. Among the many putative biological targets for imaging cancer, angiogenesis, apoptosis, signal transduction, and metabolic pathways have been the subject of intense research [5, 7, 12]. Conventional anatomic [13, 14] and some molecular imaging techniques [15–17] are currently used in the common clinical practice to study patients suffering from Pca. All these diagnostic tools have advantages and disadvantages although they play a rather limited role in monitoring men with Pca [7, 15–17]. These limitations are imputable to inability to distinguish Pca from the surrounding nonmalignant tissue. Thus, molecular imaging, providing biologically relevant information, may allow more accurate patient stratification with a more accurate therapeutic monitoring. Among all of molecular imaging modalities, PET represents a robust imaging technique because it provides noninvasive qualitative and quantitative information and requires very low levels of molecular probes to obtain images in intact living subjects [17]. Additionally, the imaging of tumor receptor poses specific challenges for the design of radiopharmaceuticals. Since most receptors have high affinities for their ligands, radiopharmaceuticals with high specific activity are essential since small molar quantities of an imaging agent may saturate a receptor and limit the ability to visualize receptor expression [17]. This paper is intended as a review of recent advances in molecular imaging of key biomarkers of androgen receptor signaling pathways and their implications for hormonal manipulation and radiation treatment in Pca.

## 2. Methods

### 2.1. Search Strategy

A computerized search was performed to identify all relevant studies in Medline up to 2013. The following search terms were used in Medline: “prostate cancer AND molecular imaging,” “prostate cancer AND radiotherapy,” “prostate cancer AND hormonal manipulation,” “PET-CT and prostate cancer,” “ProstaScint SPECT and prostate cancer,” “molecular imaging AND androgen receptor,” and “molecular imaging AND radiotherapy.” Additional articles were extracted based on recommendations from an expert panel of authors.

## 3. Imaging AR Signaling Pathway

### 3.1. 18F-Fluoro-5*α*-dihydrotestosterone (18F-FDHT)

18F-FDHT, a ligand that targets the ligand-binding domain of AR, assesses receptor occupancy but not downstream activity. Recent studies of 18F-FDHT PET in CRPC patients treated with MDV3100 found that tumors in nearly all patients showed a decrease in 18F-FDHT binding, indicating that MDV3100 can occupy the AR ligand-binding domain and preclude 18F-FDHT binding. However, these 18F-FDHT PET “responses” did not correlate with declines in serum PSA or tumor response [6]. Therefore, 18F-FDHT PET may have utility in optimizing the dose of antiandrogen required for complete blockade of androgen binding to AR, but it cannot assess AR pathway activity. Changes in AR levels may be measured by 18F-FDHT, a structural analog of 5*α*-dihydrotestosterone (DHT), in Pca patients undergoing therapy [18]. Among fluorinated androgen analogs studied in animals, 18F-FDHT uptake was reduced by about 10-fold by the coadministration of testosterone. Thus, 18F-FDHT appears to bind specifically to AR *in vivo* and to have the most favorable targeting properties for imaging among AR-binding radiotracers studied [18]. Currently, a direct biopsy of a metastatic lesion may be required to assess the AR status especially when treatment is being considered. This procedure is technically feasible, but due to its invasiveness it is not considered as a part of routine practice. Moreover, the AR status determined histopathologically in one metastasis may not be representative of all metastatic lesions. A PET ligand which provides signals able to predict and measure AR expression levels not only would have great potential in the diagnostic environment but also could have implications in tailoring the appropriate therapy and in assessing its efficacy. Preliminary clinical experience suggests that [18]F-FDHT PET is a simple way to estimate AR concentration in men suffering from CRPC metastatic disease and treated by hormonal manipulation [18]. The group of Larson, at the Memorial Sloan-Kettering Cancer Center [19, 20], confirmed that [18]F-FDHT may be a promising radiotracer for the study and imaging of AR during the progression to CRPC. This team found that [18]F-FDHT-PET detected 58 of the 59 lesions identified using conventional imaging procedures [21]. In a second study [18], at the Washington University, a team led by Dehdashti found that 10 of the 15 patients with advanced Pca and studied with [18]F-FDHT-PET, computed tomography (CT) and bone scintigraphy had tumors that took up [18]F-FDHT. In 10 patients with positive [18]F-FDHT-PET, the tumor [18]F-FDHT uptake after one single dose of flutamide was significantly decreased with a mean drop in intensity around 60% [18]. Whether this early response to antiandrogens predicts the long-term therapy response to hormonal manipulation remains unclear. However, the evidence that after flutamide treatment tumors which disappear on FDHT-PET are still visible on conventional imaging suggests that this radiotracer may be used as an early marker of tumor response. This evidence is confirmed by studies in CRPC patients treated with MDV3100 showing that tumors in nearly all patients had a decrease in 18F-FDHT binding, indicating that MDV3100 occupies the AR ligand-binding domain and precludes 18F-FDHT binding. However, these 18F-FDHT PET “responses” did not correlate with declines in serum PSA or tumor response [22].

### 3.2. Radiotracer Targeting Free Prostate-Specific Antigen

By quantitatively assessing expression of a downstream AR target gene, PET tracers targeting PSA subforms or PSMA may identify those patients whose tumors retain AR activity despite blockade of the AR ligand-binding domain and therefore would be ideal candidates for additional therapies to fully inhibit AR signaling. PSA is one of the downstream mediators whose expression reflects AR transcriptional activity in normal and cancerous prostatic cells, although additional factors regulating the PSA promoter have been identified [23]. However, even though it is used as tumor marker, PSA exhibits certain limitations. As a primary screening tool, this marker is unable to distinguish between normal pathological conditions especially in the so-called “grey zone” (PSA value from 2.5 to 10 ng/mL) [24]. For the range above 20 ng/mL or in clinical conditions characterized as androgen independent, it has been shown to be a good indicator of metastatic disease [24]. Apart from a few clinical contexts, changes in PSA levels are hard to interpret and over the last few years new PSA fractions have increasingly been used to improve the PSA diagnostic performance. Clinical and biological data suggest that tumor tissue produces greater amount of free PSA (fPSA) than normal one and this seems to improve the predictive value of this marker in detecting Pca [24]. In a recent report, a team led by Ulmert et al. [25] pointed out that the use of a monoclonal antibody, the 5A10, is conjugated with 89ZR that specifically binds fPSA and features the AR-driven prostate tumor activity. Of great interest is the fact that 89Zr-5A10 is suitable for the quantification of AR transcriptional activity in preclinical models of androgen independent models. Additionally, 89Zr-5A10 is colocalized in PSA- and AR-positive Pca models and quantitatively predicted response to antiandrogen therapy [25]. This radiotracer appears to preferentially target malignant tumor cells and therefore may become a more predictive imaging biomarker in prostate cancer [26].

### 3.3. Imaging Strategies Targeting Prostate-Specific Membrane Antigen

Another surrogate of AR transcriptional activity is the prostate-specific membrane antigen (PSMA) [26]. The molecular basis for downregulation of PSMA expression by AR may be related to the presence of an enhancer region although no androgen response elements have been identified [27]. Recent AR ChIP-Seq reveals four peaks of AR binding among multiple introns of PSMA in LNCaP [28]. Preliminary data indicated that PSMA, a membrane glycoprotein, was specifically expressed on Pca cells as a noncovalent homodimer and, for this reason, was regarded as specific for prostate tissue [29]. More recently, its expression has been documented in a few other tissues reducing its specificity for prostate [29]. Although PSMA is no longer considered prostate specific, the literature indicates that this glycoprotein may be useful in nuclear medicine for imaging benign and malignant prostate tissue [29]. Little is known about its function in the biology of normal and pathological conditions although some evidence suggests a role of this glycoprotein as oncosuppressor. This hypothesis is supported by the evidence that PC3 cells, transfected with full length PSMA cDNA and then orthotopically implanted into nude mice, gave rise to lower tumor volumes with reduced incidence of metastases with respect to mice implanted with wild-type PC3 cells [30]. Interestingly, its activity and expression are increased as tumor becomes more androgen independent [31]. This clearly indicates that there is a link between PSMA and androgen independent phenotype [30]. From a clinical point of view this may have a significant impact on the possibility to select men with CRPC who can be at risk of resistance to hormonal manipulation and/or radiation therapy. This statement may be confirmed by reports indicating that changes in PSMA expression can also serve as a noninvasive marker for imaging AR signaling and monitoring response to conventional treatments [29, 32]. Additionally, the molecular imaging strategy using tracers directed towards PSMA could also have a more significant clinical impact considering that PSMA is upregulated in response to antiandrogen therapy [33]. This may have important therapeutic implications since a toxin-conjugated PSMA-targeted mAb could be an effective combination therapy with antiandrogens. Indeed, J591 has been adapted for radioimmunotherapy, and Ab-drug conjugates and therapeutic doses are well tolerated in patients [34, 35]. A well-known Food and Drug Administration (FDA) approved that agent targeting PSMA is capromab pendetide (ProstaScint) [36]. It consists of a murine monoclonal antibody (mAb) (mAb 7E11) labeled with 111In [36]. The overall diagnostic performance of ProstaScint for detecting Pca is variable in function according to different reports with an average sensitivity, specificity, and positive and negative predictive value of 60%, 70%, 60%, and 70%, respectively [7, 36]. The relatively disappointing results of these studies are partially due to the murine monoclonal antibody properties. This antibody binds to the internal epitope of PSMA and therefore shows limited effectiveness in targeting viable prostate tumor cancer cells. Thus, only cells with damaged cell membranes bind mAb 7E11, explaining the decreased overall performance of this imaging modality [36]. More recently, radiolabeled mAbs that bind the extracellular PSMA domain have been developed [37]. The second-generation of antibodies targeting PSMA provide higher performance than capromab pendetide. Among these, the monoclonal antibody J591 [38] has provided outstanding results in imaging bone metastatic disease. J591 has been extensively utilized and evaluated in different preclinical models, demonstrating higher specificity in identifying tumor tissue [38]. Clinical trials, using J591 labeled with 99mTc, demonstrated the detection of distant metastases, including bone metastases, in patients with CRPC [39]. Several other novel mAbs targeting PSMA have been developed for molecular imaging. Among these, four new IgG mAbs (3/F11, 3/A12, 3C6, and 3/E7) with strong affinity for three different extracellular PSMA epitopes [40, 41] have been developed. The 3/A12 antibody labeled with 64Cu (64Cu-3/A12) demonstrated a good tumor-to-background ratio in preclinical models [42]. However, the slow tumor uptake and plasma clearance of mAbs in men necessitate the use of long-lived radioisotopes for imagining at the expense of increased radiation dose to the patients [43]. A further disadvantage is the need to bring the patient for a second visit several days after injection for imaging [43]. Low-molecular-weight radiopharmaceutical agents have better pharmacokinetic properties than radiolabeled mAbs [43]. These low-molecular-weight molecules have encouraged the generation of a new class of PSMA targeting molecules for single-photon emission computed tomography (SPECT) and PET imaging [31, 44–46]. Among these, the PSMA tracer N-[N-[(S)-1,3-dicarboxypropyl]-carbamoyl]-S-[11C] methyl-l-cysteine (DCFBC) has been developed for PET imaging [47–50]. This radiotracer has been used successfully for imaging preclinical xenograft models expressing PSMA antigen [47–50]. More recently, Mease and colleagues [51] and Lapi and coworkers [52] labeled DCFBC and phosphoramidates compounds, respectively, with 18F. Biodistribution and imaging studies showed a PSMA-expression dependent tumor uptake of this radiotracer with higher uptake in PSMA expressing tumor cells [51, 52]. Other promising small-molecule inhibitors for Pca imaging are MIP-1072 and MIP-1095 [53]. These agents are urea based compounds and exhibit high affinity for PSMA [29, 53]. When labeled with 123I, they have been successfully used as radiotracers with SPECT/CT in human Pca xenografts [53]. Finally, limited experience with thermally cross-linked SPION (TCL-SPION), able to both detect Pca cells and deliver targeted chemotherapeutic agents directly to Pca cells, has been reported [47–50]. Differential uptake of the TCL-SPION-Apt bioconjugates by PSMA-expressing LnCaP cells or non-PSMA-expressing PC3 cells was documented confirming that TCL-SPION-Apt bioconjugates can differentially target PSMA-expressing Pca cells [47–50]. This agent, in addition to the efficient identification of Pca cells *in vivo* by magnetic resonance imaging (MRI), is able to selectively deliver cytotoxic drugs to the tumor tissue, providing an excellent compromise in terms of diagnostic and therapeutic capabilities [47–50].

## 4. Conclusions

New imaging modalities allowing the investigation of molecular events in terms of the spatiotemporal dimension may be useful to follow the intracellular signaling pathways both in the tumor itself as well as in the surrounding normal tissues. Molecular imaging comprises a cluster of technologies allowing the measurement of biological events that are relevant for the understanding and the monitoring of prostate cancer, especially when it becomes resistant to treatments. Each different imaging modality presents its unique set of advantages and disadvantages in terms of sensitivity, resolution, and type of information provided. To overcome these drawbacks, innovative technologies, allowing the integration of different imaging modalities, have been developed. Further advances are also expected to be in the way tracers are conceived to widen the number of biological events that can be studied and monitored by molecular imaging. Among them, PET-based and, to a lesser extent, MRI-based technologies are promising modalities which have opened up new avenues for visualizing and understanding the biological changes occurring in patients that do not respond to hormonal and radiation treatment. Obviously, the information obtained in such manner could not be sufficient to unravel the molecular pathways that govern the mechanisms involved in the resistance to treatments but might represent a powerful tool for visualizing and understanding differences in the cancer biology that become manifested between a responding patient and a nonresponding patient. Hopefully, the knowledge of critical molecular events involved in these biological processes will allow us to identify unique signatures useful to inspire development of new therapeutic strategies for overcoming the problem of resistance to conventional treatments.
